# Incidence and Clinical Significance of *De Novo* Donor Specific Antibodies after Kidney Transplantation

**DOI:** 10.1155/2013/849835

**Published:** 2013-11-21

**Authors:** Sophia Lionaki, Konstantinos Panagiotellis, Aliki Iniotaki, John N. Boletis

**Affiliations:** ^1^Nephrology Department and Transplantation Unit, Laiko Hospital, 17 Ag. Thoma Street, 11527 Athens, Greece; ^2^National Tissue Typing Center, General Hospital of Athens “G .Gennimatas”, Greece

## Abstract

Kidney transplantation has evolved over more than half a century and remarkable progress has been made in patient and graft outcomes. Despite these advances, chronic allograft dysfunction remains a major problem. Among other reasons, *de novo* formation of antibodies against donor human leukocyte antigens has been recognized as one of the major risk factors for reduced allograft survival. The type of treatment in the presence of donor specific antibodies (DSA) posttransplantation is largely related to the clinical syndrome the patient presents with at the time of detection. There is no consensus regarding the treatment of stable renal transplant recipients with circulating *de novo* DSA. On the contrast, in acute or chronic allograft dysfunction transplant centers use various protocols in order to reduce the amount of circulating DSA and achieve long-term graft survival. These protocols include removal of the antibodies by plasmapheresis, intravenous administration of immunoglobulin, or depletion of B cells with anti-CD20 monoclonal antibodies along with tacrolimus and mycophenolate mofetil. This review aims at the comprehension of the clinical correlations of *de novo* DSA in kidney transplant recipients, assessment of their prognostic value, and providing insights into the management of these patients.

## 1. Introduction

Despite all advances in the development of effective immunosuppressive regimens in kidney transplantation, chronic allograft dysfunction remains a major problem [1]. Humoral immune response contributes to the development of this entity. Pretransplantation unsensitized kidney transplant recipients may develop *de novo* antibodies against donor human leukocyte antigens (HLA) or non-HLA as are the polymorphic MHC system class I chain-related gene A (MICA) molecules [2]. *De novo* formation of donor specific antibodies (DSA), directed against HLA, has been recognized as one of the major risk factors for reduced allograft survival. First observation was in 2002, when anti-HLA antibodies were shown to appear 6 months to 8 years before graft failure in a serial longitudinal study [3]. Now, we have substantial evidence, showing that formation of *de novo* DSA after kidney transplantation is associated with antibody-mediated graft injury that may lead to graft failure [4, 5]. Anti-HLA class II DSA are considered the predominant *de novo* produced antibodies posttransplantation in unsensitized pretransplantation renal transplant recipients [6–8]. The introduction of more sensitive and specific assays [9], as well as serial evaluation of multiple samples [10] from the same patient, allowed the detection of DSA after transplantation and comprehend of their interference to graft dysfunction. The aim of this paper is to review the incidence of *de novo* detected anti-HLA and anti-MICA DSA after kidney transplantation, underlining their clinical impact and pathologic correlations and assess their prognostic ability to transplantation outcome. Insights into the management of patients with posttransplant formation of DSA are also within the scope of this review.

## 2. *De Novo *Developed Posttransplantation Donor Specific Antibodies

Patient's exposure to “nonself-” HLA molecules as after blood transfusion, pregnancy, or organ transplantation can lead to the development of anti-HLA antibodies [11, 12]. Thus, a transplant candidate may present with preformed anti-HLA antibodies, while being in the waiting list. The antibodies that do not preexist but develop after transplantation and are directed against foreign graft HLA are considered as *de novo* anti-HLA DSA. The distinction as donor specific is crucial, when considering clinical relevance of anti-HLA antibodies, as the DSA are for the graft. However, the Mayor Histocompatibility (MHC) system is highly polymorphic, with “private” epitopes that characterize each specific allele and “public” epitopes occurring between alleles, not only in the same locus, but also in different loci [13]. Humoral sensitization on the other hand is a matter of anti-HLA antibodies that recognize epitopes expressed on specific HLA molecules. An epitope is defined as the physical area of an antigenic molecule that an antibody binds to. In the case of proteins, epitopes are defined by the tertiary conformation of amino acid sequences. As a result, the primary sequence of amino acids of a protein does not necessarily define an epitope. HLA epitopes are structurally defined with the usage of an algorithm [14] which is important when classifying a given anti-HLA antibody as DSA. Another crucial characteristic is the difference between the antigenicity of epitopes (i.e., the reactivity with the anti-HLA antibody) and the immunogenicity of epitopes (i.e., the capacity of inducing anti-HLA antibody) [15]. Yet, partial denaturation of antigens may lead to false positive results. Thus, it is essential to elucidate the difference between HLA epitopes and antigens in the light of understanding humoral immune response in transplant recipients.

The MICA genes were first described in 1994 [16]. They are located in the 46 kb centromeric to HLA-B region and encode molecules similar in conformation to HLA class I proteins. MICA genes are polymorphic and several studies have found that immune response against MICA may correlate with a decrease in graft survival after transplantation. MICA antigens are expressed in epithelial cells [17], in fibroblasts, in endothelial cells, and in monocytes and dendritic cells [18, 19]. Lymphocytes are devoid of MICA and thus cross-matching with lymphocytes obtained from the blood does not work for the detection of antibodies against MICA. Only activated lymphocytes have been reported to express MICA [20], indicating them as danger signals in helping activating innate immunity through binding of NKG2D on natural killer cells and certain T cells [21]. A collaborative study of more than 200 patients and their donors, with typing for MICA alleles by sequenced-based typing to determine antibodies against MICA, showed that antibodies against MICA are donor specific [22]. Anti-MICA antibodies also recognize nonself-“private” and “public” epitopes on MICA molecules [22].

## 3. Incidence of *De Novo* Donor Specific Antibodies

Several investigators have searched the incidence rate of *de novo* formation of anti-HLA DSA among kidney transplant recipients. Nevertheless, there is considerable variation in the reported rates, basically related to the diversity of methods used to detect anti-HLA antibodies [2, 23]. During the past decade, HLA antibody tests have moved from CDC to solid-phase assays, which show increased sensitivity and specificity to detect HLA antibodies [24]. Using Luminex technology one can detect and define low levels of these antibodies, which has been a substantial help in clinical practice. Early in 2002, antibodies directed against HLA were shown to appear 6 months to 8 years before graft failure of kidney transplants in a serial longitudinal study [3]. Following this observation, another study showed that antibodies appear to HLA class I and HLA class II, within approximately 2.7 to 3.9 years, respectively, before failure [6]. We know now that the incidence of anti-HLA antibodies developing 6 months after transplantation is roughly the same as after 10 years [25]. In a prospective design study, Terasaki and Cai evaluated 2231 patients and found that 21.4% of them were positive for anti-HLA antibodies 1 year after transplantation [12]. Lachmann et al. [9] studying a large cohort of 1014 kidney transplant recipients from deceased donors, monitored in a cross-sectional manner for the development of anti-HLA antibodies, found that 29% of them became positive and 31% of these antibodies were DSA. Another study of 72 patients, who were also negative for anti-HLA antibodies before transplantation, showed that 22.2% of them developed antibodies after transplantation, while 75% of them had DSA [26]. Wiebe et al. [27] evaluating a cohort of low risk patients found that 15% of them developed *de novo* DSA, in a mean follow up time of 6.2 ± 2.9 years. The mean time to development of these antibodies was 4.6 ± 3.0 years. In a retrospective analysis of 505 patients Willicombe et al. reported a rate of *de novo* production of DSA in 18.2% of patients [5]. The mean time to detection of DSA after transplantation was 9.98 ± 12.48 months [5]. In our center [8], periodical screening of 597 kidney transplant recipients revealed that 15.4% of them produced DSA after transplantation, with similar rates recorded between individuals sensitized not against the donor before transplantation and those without anti-HLA antibodies. Another recent study of 82 previously negative pediatric patients showed that 23% of them developed DSA in a mean follow-up time of 4.3 years [28]. The mean time to appearance of *de novo* DSA in this population was 24 months after transplant. Wang et al. measured the incidence of *de novo* DSA in the serum of 620 kidney recipients one year after transplantation and found that 7.3% of them had developed anti-HLA antibodies, with 84.4% of them being DSA [29]. Likewise, 32% of previously nonsensitized patients developed *de novo* DSA in the study by Gingu et al. [30]. Everly et al. [31] reported that 11% of the patients without detectable DSA at the time of transplantation will have detectable DSA 1 year later, and over the next 4 years, the incidence of *de novo* DSA will increase to 20%. After *de novo* DSA development 24% of the patients will fail within 3 years [31].

Antibodies against MICA antigens have been found in transplant patients [32, 33] and in about 10% of the patients in the waiting list for a first kidney [22]. When these antibodies are donor specific they correspond to mismatched MICA epitopes [22]. According to Zou and Stastny, about 20% of kidney transplant recipients may present with anti-MICA antibodies [22]. The authors also report a higher frequency, accounting for 30% of the patients who have rejected a previous transplant [22]. Another study, which employed integrative genomics analysis of ProtoArray data, showed that antibody responses against MICA antigens are modulated after transplantation, irrespective of the graft rejection, and may be very high at the time of humoral rejection, or simply elevated in cellular rejection [34]. They report that 73% of the patients showed an increase in MICA specific antibody response after transplant, regardless of the presence or absence of biopsy proven graft rejection [34]. Furthermore they found that MICA is preferentially localized to the glomerulus [34].

## 4. Clinical Correlations of *De Novo* Donor Specific Antibodies

Recent studies have provided substantial evidence that the development of *de novo* DSA is associated with antibody mediated injury and allograft failure [4, 5]. Studies from the past also support a significant role for anti-HLA antibodies in chronic allograft rejection [35]. Posttransplant production of anti-HLA antibodies, especially in the presence of circulating antibodies to donor HLA antigens, is highly associated with the incidence of acute rejection and graft loss [3, 6, 36, 37]. Patients with *de novo* DSA may be classified according to the clinical syndrome they present with at the time of detection, as follows: (i) acute allograft dysfunction, that is, patients with a rise in serum creatinine ≥25% from baseline in ≤2 months. In this group of patients the onset of *de novo *DSA was shown concurrent with the onset of clinical dysfunction. (ii) Indolentallograft dysfunction, that is, patients with graft dysfunction (proteinuria ≥0.5 g/day or increase in serum creatinine ≥25% in >2 months). This group includes all patients in whom the onset of *de novo* DSA preceded the start of clinical dysfunction by an average of 9 months [27], (iii) stable renal function in the allograft, including patients with no graft dysfunction, in whom DSA were detected by routine surveillance [27]. Results from a study which categorized a cohort of 315 low risk patients using the above scheme showed that independent predictors of *de novo* DSA production were HLA-DRB1 (OR: 5.66, *P* < 0.006) and nonadherence (OR: 8.75, *P* < 0.001). Specifically, nonadherence was documented in 100% of the acute dysfunction group, in 53% of the indolent dysfunction *de novo* DSA group, and in only 6% of the stable function group. In the group with acute dysfunction, the onset of *de novo* DSA was essentially concurrent with the onset of clinical dysfunction and the mean serum creatinine at the time of kidney biopsy was 5.57 mg/dL [27]. Graft loss occurred in 22/315 patients during the study period and 14 of them (63.7%) had *de novo* DSA [27]. In the study by Willicombe et al. *de novo* production of DSA, of any specificity, was found to be associated with acute mediated rejection (AMR) and transplant glomerulopathy. Only HLA-Cw DSA were found not to be significantly associated with allograft failure [5]. The major risk factor for the development of these DSA posttransplant, revealed from this study, was the higher mean HLA mismatch [5]. More detailed analysis showed that the antigen mismatches were associated with the anti-HLA DQ DSA, while patients mismatched at the HLA-DR locus were at significant risk for the developing DQ DSA (*P* = 0.0021) [5]. Thus, there is an enhanced immunogenicity with mismatching at both the HLA-DR and HLA-DQ loci, which is associated with increased production of *de novo *anti-HLA DQ DSA [5]. The high incidence of anti HLA DQ DSA is probably related to the high number of polymorphic epitopes that are expressed on both *α* and *β* chains of the HLA-DQ molecule [38].

In our experience [8], *de novo* appearance of DSA was significant in that anti-HLA class II DSA, mostly directed against HLA-DQ molecules, were predominant in HLA class II incompatible grafts (Table 1). Moreover, recipients of HLA-class II incompatible grafts developed DSA more frequently than those receiving HLA-class II compatible (17.9% versus 7.9%, *P* = 0.003) [8]. Over the follow-up time, 48/597 (8%) patients lost their graft and 28/48 (58.3%) of these had *de novo* formation of DSA. The presence of detectable anti-HLA antibodies, either DSA or non-DSA, was the only independent predictor for graft loss in the study coming from our center. Hazards ratios for DSA positive and DSA negative patients were found in 22.54, 95% CI: 6.69–75.89, *P* < 0.001 and 5.94, 95% CI: 1.67–21.06; *P* = 0.006, respectively [8]. A retrospective study [28], evaluating the *de novo* formation of DSA in a pediatric population of 82 patients, showed that renal function, measured as serum creatinine, was significantly different between patients with and without *de novo* formation of DSA. Specifically, patients who did not develop anti-HLA antibodies, and those with non DSA had comparable serum creatinine levels at discharge, and throughout the follow-up time (mean time 4.3 years). Conversely, a significant increase in creatinine levels was observed in the DSA group at the end of follow-up period, when compared with values at discharge and at the time of first DSA appearance [28]. Interestingly, Lee et al. studying serial sera collected in a period of 17 years from two groups of patients, one whose allograft failed due to chronic rejection and a control group consisting of patients with functioning grafts matched by transplant date, found that DSA appeared in 96% of the patients with graft failure versus 48% of the controls [42]. Importantly, this study provided clear evidence that time to development of posttransplant antibody is a critical factor in determining allograft survival. According to these findings, antibodies which were developed within a year after transplantation resulted in graft failure in a mean time of 5.1 years [42]. In contrast, antibodies, which were formatted after the first year, were associated with a slow rate of failure and 80% of patients had functioning grafts one decade after transplantation [42]. This difference was mostly attributed to the condition of the graft and probably the response of the host to the graft. It is possible that antibodies forming within the first year react rapidly on the endothelium initiating a cascade of events leading to rejection. In the same study it was shown that HLA class I DSA are produced sooner (median time to detection 6.6 months) and are associated with rapid graft loss, while class II DSA occur later (median time to detection 12.5 months) and may be associated with chronic transplant glomerulopathy [42]. Huang et al. [43] monitoring performed and *de novo* HLA DSA found the incedence of acute rejection in 34%, 48%, and 70% for patients with no DSA, with performed DSA and with *de novo* DSA, respectively. Notably, in all recipients with *de novo* DSA and rejection the first rejection episode preceded or was concurrent with the emergence of *de novo* DSA. Likewise DeVos et al. [44] reported that patients who developed DSA after transplant had increased rate of acute rejection episodes, higher serum creatinine, and worst graft survival. Moreover patients with persistent DSA had increased rates of rejection and worst renal function [44]. However, a prospective DSA screening protocol failed to identify patients at risk for acute rejection or poor short term graft outcomes [45]. Specifically, although DSA was detected in 27% of all patients by protocol or indication searching, and patients with DSA were significantly more likely to have experienced acute rejection compared with those without DSA, only 3 out of 19 with DSA had DSA detected before the rejection episode [45]. 

MICA antibodies are also becoming increasingly recognized as critical in the pathogenesis of organ allograft outcomes. Prospective studies of patients with MICA antibodies have shown that they experience lower allograft survival accounting for 83% versus 94% for patients with HLA antibodies or 96% for those without HLA antibodies (*P* = 0.0005, *P* = 0.0004, resp.) [46]. Notably, the multivariate analysis from that study revealed hazard ratios for patients with MICA antibodies as high as 6.1 opposed to 3.6 for patients with HLA antibodies (*P* < 0.00001) [46]. An earlier retrospective study had identified MICA in kidneys which had been rejected [47]. They were also found in the sera of patients who eventually experience graft failure at a higher frequency than in those who had functioning grafts [48]. Moreover, MICA antibodies were found before transplantation in about 25% of the 85 patients in the waiting list [44] and associated with hyperacute rejection and in the absence of HLA antibodies [48]. Several studies have shown that kidney allograft recipients undergoing both acute and chronic rejection may have measured antibodies against MICA antigens [32, 49–52].

## 5. Pathologic Correlations

A key to understanding the effects of antibody-mediated graft damage is to define the relationship between donor specific antibody in the recipients' sera and the histopathological lesions in their grafts. The current Banff criteria define the diagnosis of AMR as the presence of DSA along with certain histological changes [48] including the C4d deposition [50]. In this regard, Hidalgo et al. [53] studied the frequency of *de novo* DSA in the sera of patients and their associations with specific histologic lesions and prognosis. They found that *de novo* DSA were more frequent in patients having late biopsies (34%) versus early biopsies (4%) [53]. Microcirculation inflammatory changes, such as glomerulitis, or capilliritis, and damage such as glomerulopathy, or capillary basement membrane multilayering and C4d staining were associated with *de novo* DSA [53]. Thus, in late biopsies, *de novo* DSA detection is frequent and associated with microvascular lesions but not with scarring lesions. This study, performed in late biopsy population, indicates that *de novo* DSA stresses microcirculation in the allograft, causing these kidneys to present with clinical indications for biopsy at a median of about 6 years after transplant. Weibe et al. on the other hand, studying 315 consecutive renal transplants without pretransplant DSA, with protocol and for cause biopsies, correlated the patients' clinical phenotypes by graft function at the time of *de novo* DSA detection. They found that 0–6-month clinical rejection episodes (borderline or Banff 1A/1B cellular rejections) occurred more commonly in the *de novo* DSA group compared with the patients without *de novo *DSA (28% versus 13%, *P* = 0.015). In addition, despite a median acuteglomerulitis score of zero in both groups, the *de novo* DSA group had significantly higher peritubular capillaritis scores in 0–6-month clinical rejection biopsies compared to the group without *de novo* DSA (2 versus 1, *P* = 0.049). Moreover, both the clinical rejection frequency and the grade of capillaritis were higher independent of adherence in the *de novo* DSA group [27]. Despite the widespread use of C4d staining in the clinical management of kidney transplant recipients [54–59], over time it has become clear that C4d is neither completely specific nor sufficiently sensitive for the diagnosis of AMR [54–56, 60]. The current Banff diagnostic criteria [57] however require positive C4d peritubular capillary staining for a definite diagnosis of AMR, which might exclude some patients from such a specific diagnosis. Interestingly, C4d-negative AMR, a clinical entity which emerged in 2009 [56], was shown to be associated with microcirculation changes and the presence of anti-HLA class II DSA. As predictors of progression to chronic mediated rejection, these findings confirmed the notion that independently of C4d positivity, C4d-negative kidneys could share features of antibody-mediated injury, and C4d staining alone may not be sensitive enough to establish a diagnosis of acute mediated rejection. Another study demonstrated that high endothelial-specific gene expression in biopsies from kidney transplant recipients with DSA but without C4d, indicating ongoing antibody-mediated damage [56]. Most cases of C4d-negative AMR tend to occur more than 1 year after transplantation and represent chronic or acute-on-chronic AMR episodes [56].

## 6. The Methods of Measuring DSA in Serum and Diagnosis of DSA in Serum and Diagnosis of DSA-Associated Graft Rejection

Although the complement-dependent cytotoxicity (CDC) [61] crosshatch has been the gold standard assay for many years in kidney transplantation, the need for alternative HLA antibody screening was also clear. The target in the CDC assay is the lymphocyte, and thus not only HLA molecules but also other unrelated cell membrane components may be targets for antibody reactivity. Moreover, autoantibodies, immune complexes, and immunoglobulin allotypes may also interfere in this assay [62]. Because the assay is based on complement activation, HLA specific IgG antibodies, not able to fix complement, such as IgG2 and IgG4, cannot be detected. This problem was solved by the development of solid phase assays using isolated HLA molecules as targets for antibody detection. They are based on ELISA or fluorescence and can detect both complement fixing and noncomplement fixing IgG antibodies [63]. A positive reaction in this occasion is by definition caused by the reactivity with an HLA class I or a class II molecule, and not with another unrelated cell membrane molecule. 

The ELISA assay was developed first, in which HLA class I and HLA class II molecules were used as target molecules. Later studies showed that kidney transplant recipients who were transplanted with a negative CDC crossmatch with both HLA class I and HLA class II antibodies present before transplantation with this assay had a significantly poorer graft survival [63] compared with patients without HLA antibodies. Yet, patients with HLA class I or HLA class II antibodies did not experience this worst graft survival. This effect was attributed to donor specific antibodies as the impact on graft survival was shown greater with the number of HLA mismatches between donor and recipient [61]. Subsequently, Lefaucheur et al. showed a significantly lower graft survival in patients with DSA compared with patients without DSA [62, 63]. In this regard, when the presence of DSA was associated with the occurrence of AMR graft survival was worse in the DSA group, while in the absence of AMR graft survival was similar to that of patients without DSA. Therefore, although the DSA detected by ELISA are a risk factor for some patients, it is not feasible to assign the risk on a specific patient [56]. More recently, assays based on antibody reactivity against HLA molecules attached to Luminex beads are used. The availability of single HLA antigen beads facilitates the determination of the antibody specificity enormously compared to previous panel analyses [64]. There are several retrospective studies showing that the presence of DSA is associated with a significantly decreased graft survival even if no AMR takes place [64–66]. Yet, donor HLA class II specific antibodies detected by Luminex were shown to be clinically relevant with a positive B-cell crossmatch [67]. Other studies reported that DSA detected in Luminex are irrelevant in patients transplanted with a negative CDC crossmatch [68, 69] or with a negative flow cytometric crossmatch [69]. Incidence of rejection, renal function and graft survival were found similar between patients with and without DSA BY Luminex. 

Late AMR is a major cause of late kidney transplant failure and major assistance in its understanding was provided by the 1997 Banff classification. It was created basically in order to classify rejection prior to the meeting in 2001, which further defined pathological classification of AMR [49]. Incidence of AMR has been reported as 0–8% in renal transplant recipients in larger centers largely due to increased recognition, detection of DSA, retransplanted patients, and increase in positive crossmatch and ABO incompatible transplantation for highly sensitized patients. A few studies have indicated C4d staining is around 93–96% specific but 31–95% sensitive [49]. Lately quantification of DSA has been introduced as a comarker of AMR to be taken into consideration in diagnosis and treatment. After the Banff meeting in 2001 it was determined that AMR has 3 cardinal features upon biopsy findings: (i) acute tubular injury; neutrophils and/or mononuclear cells in peritubular capillaries and/or glomeruli, and/or capillary thrombosis; or arteritis/fibrinoid necrosis in the intima along with intramural/transmural arterial inflammation. (ii) C4d evidence for antibody action and/or immunoglobulin in peritubular capillaries, immunoglobulin and complement in arterial fibrinoid necrosis. (iii) anti-HLA antibody (DSA) circulation in serum or other anti-donor endothelial antigens. 

After the Banff meeting in 2009, positive C4d deposition without evidence of rejection was added to criteria suggesting antibody-mediated changes. Several studies had reported C4d deposition in biopsies without evidence of rejection [56]. In protocol biopsies from ABO incompatible grafts, 21/37 had C4d deposition without evidence of AMR lesions or T-cell-mediated rejection, which can suggest accommodation. Another addition to Banff criteria indicating antibody-mediated changes was determined to be positive C4d with presence of circulating anti-donor antibodies (no signs of acute or chronic T-cell-mediated rejection or AMR/no ATN-like minimal inflammation) [70]. 

## 7. Management of the Patient with *De Novo* DSA after Kidney Transplantation

Patients with *de novo* DSA may present with a spectrum of clinical syndromes and various pathological features. The clinical phenotypes of the patients who develop DSA after kidney transplantation vary significantly, from acute allograft dysfunction to stable renal function [27]. In this regard, the best therapeutic approach for the kidney transplant recipient with *de novo* DSA will depend on the clinical picture at the time of detection. Between episodes of acute AMR and late chronic AMR there is a dynamic and progressive process of injury and repair standing, which ultimately contributes to failure of the allograft. Therefore, it is believed that the most important clinical criterion of which protocol to use is the rapidity of onset of graft dysfunction. Therapeutic protocols include removal of the antibodies by plasmapheresis (or immunoadsorption), suppression of antibody production by intravenously administered immunoglobulin (IVIG), depletion of the antibody-secreting plasma cells with anti-CD20 treatment with rituximab, along with tacrolimus and mycophenolate mofetil. Most recent protocols employ Campath-1H and Bortezomib [70]. Patients with *de novo* formation of DSA after transplantation are treated by the clinical syndrome they present with at the time of detection: (i) Acute allograft dysfunction with histological evidence of antibody mediated injury (C4d+) with minimal pathologic features, acute tubular necrosis-like changes, are treated with a combination of pulse methylprednisolone, a course of plasma exchange therapy, 6–8 sessions, IVIG, and one pulse of rituximab, 375 mg/m^2^. However, patients with more severe clinical picture, that is rapid deterioration of renal function and diffuse C4d+ staining, evidence of capillary and/or glomerular inflammation with thrombosis require therapy with longer courses of plasma exchange, 8–10 sessions, followed by IVIG and rituximab. The new agent, which has emerged in the treatment of AMR episodes, is bortezomib [71]. It has been shown effective in reducing anti-HLA antibody levels and reversing both AMR episodes in substantial numbers of treated patients.

In both occasions, formation of *de novo* DSA is typically documented in the light of worsening of renal function and typically at the same time with the biopsy showing acute AMR. It is essential to note that for this group of patients institution of therapy should be rapid to avoid irreversible graft loss. Despite the fact that a biopsy result is not a requirement for institution of treatment, a graft biopsy is required to avoid treating patients with advanced degrees of interstitial fibrosis and tubular atrophy who are unlikely to benefit. (ii) Indolent allograft dysfunction represents a slower, gradual decline of renal function, which occurs without acute compromise of renal function or significant proteinuria and cannot be explained by any other cause. Kidney transplant recipients who develop *de novo* DSA often show pathologic features of indolent and slowly progressive microvascular abnormalities. Donor specific antibodies, formed *de novo*, bind to allogenic targets expressed by graft endothelium activate the system of complement system and modulate the biology of rejection. The appearance of these antibodies results from inadequate immunosuppression and thus prevention is synonymous with sufficient immunoregulation and/or enhancing of the level of as needed. (iii) Detection of *de novo* DSA in a routine test in patients with stable allograft function represents a step behind in the continuum of the natural history of acute AMR. It is largely unknown how to treat these patients. A closer monitoring of these patients, in addition to the advancement of immunosuppressive therapy, which typically includes tacrolimus and mycophenolate mofetil is generally suggested. We tend to maintain higher trough level of tacrolimus in such patients (>6 ng/dL) and usually administer 1.5–2 g of mycophenolate mofetil per day, depending on the body weight. However, recent investigation has shown other potential pathways that might be beneficial in such patients. For instance, the effect of the proteasome inhibitor bortezomib was evaluated as a desensitization strategy in recent study [72]. They administered one cycle of bortezomib (1.3 mg/m^2^ × 4 doses), used as the sole desensitization therapy. Bortezomib treatment did not significantly decrease DSA within the 150-day posttreatment period in any of the 4 patients [72].

## Figures and Tables

**Table 1:** Studies searching the incidence of *de novo* anti-HLA DSA and their impact on graft survival.

Author Publication year	Cohort size, *N*	Incidence of *de novo* anti-HLA DSA	*De novo* anti-HLA abs, class, DSA frequency	Followup (years)	Incidence of AMR in pts. with *de novo* anti-HLA Abs	Incidence of GF in total	Incidence of GF in pts. with *de novo* anti-HLA and anti-HLA DSA
Worthington et al. 2003 [6]	76	10.5%	Class I, 7.9% Class II, 6.6% Class I and II, 1.3%	10		23.7%	91.7%
Hourmant et al. 2005 [23]	1229	5.5%	Class I, 0.1% Class II, 5.4%	5	8%	8.2%	16.8% 4.8%
Terasaki and Cai 2005 [37]	1564			2		8%	16.7%
Zhang et al. 2005 [39]	49	22.4%	Class I, 10.2% Class II, 6.1% Class I + II, 6.1%	2	26.7%		
Mihaylova et al. 2006 [26]	72	16.7%	Class I, 9.7% Class II, 5.6% Class I + II, 1.4%	1–5	8.3%	18%	56.25%
Mao et al. 2007 [40]	54	27.8%		5		46.3%	65.6% 40.6%
Lachmann et al. 2009 [9]	1014	9.2%	Class II, 6%	5.5	3.6% 2.4%	20.9%	14.7%
Ntokou et al. 2011 [8]	597	15.4%	Class I, 3.2% Class II, 11.4% Class I + II, 0.8%	1.2–10	6% 3.7%	8%	15.6% 9.7%
Wang et al. 2012 [29]	620	6.2%	Class I, 1.5% Class II, 3.9% Class I + II 0.8%, DSA	5		18.4%	60% 63.2%, DSA
Ginevri et al. 2012 [28]	82	23.1%	Class I 2.4%, DSA Class II 13.4%, DSA Class I + II 7.3%, DSA	4.3	40% 36.7%, DSA		13.5%, DSA
Alberu et al. 2012 [41]	53	32%	Class I 20.7%, DSA Class II 7.5%, DSA Class I + II 3.8%, DSA	2	5.7%, DSA	9.4%	23.5%, DSA
Wiebe et al. 2012 [27]	315	14.9%	Class I 0.9%, DSA Class II 10.2%, DSA Class I + II 3.8%, DSA	2.9 ± 6.2	5.3%, DSA	7%	13.6%, DSA
Willicombe et al. 2012 [5]	505	18.2%	Class I 5.5%	5	30.6%, DSA		14.4%, DSA

Abs: antibodies; DSA: donor specific antibody; HLA: human leucocyte antigen; AMR: antibody-mediated rejection; GF: graft failure.

## References

[1] Meier-Kriesche H-U, Schold JD, Srinivas TR, Kaplan B (2004). Lack of improvement in renal allograft survival despite a marked decrease in acute rejection rates over the most recent era. *American Journal of Transplantation*.

[2] Akalin E, Pascual M (2006). Sensitization after kidney transplantation. *Clinical Journal of the American Society of Nephrology*.

[3] Lee P-C, Terasaki PI, Takemoto SK (2002). All chronic rejection failures of kidney transplants were preceded by the development of HLA antibodies. *Transplantation*.

[4] Terasaki PI, Ozawa M, Castro R (2007). Four-year follow-up of a prospective trial of HLA and MICA antibodies on kidney graft survival. *American Journal of Transplantation*.

[5] Willicombe M, Brookes P, Sergeant R (2012). De novo DQ donor-specific antibodies are associated with a significant risk of antibody-mediated rejection and transplant glomerulopathy. *Transplantation*.

[6] Worthington JE, Martin S, Al-Husseini DM, Dyer PA, Johnson RWG (2003). Posttransplantation production of donor HLA-specific antibodies as a predictor of renal transplant outcome. *Transplantation*.

[7] Issa N, Cosio FG, Gloor JM (2008). Transplant glomerulopathy: risk and prognosis related to anti-human leukocyte antigen class II antibody levels. *Transplantation*.

[8] Ntokou I-SA, Iniotaki AG, Kontou EN (2011). Long-term follow up for anti-HLA donor specific antibodies postrenal transplantation: high immunogenicity of HLA class II graft molecules. *Transplant International*.

[9] Lachmann N, Terasaki PI, Budde K (2009). Anti-human leukocyte antigen and donor-specific antibodies detected by luminex posttransplant serve as biomarkers for chronic rejection of renal allografts. *Transplantation*.

[10] Einecke G, Sis B, Reeve J (2009). Antibody-mediated microcirculation injury is the major cause of late kidney transplant failure. *American Journal of Transplantation*.

[11] Halloran PF (2003). The clinical importance of alloantibody-mediated rejection. *American Journal of Transplantation*.

[12] Terasaki PI, Cai J (2005). Humoral theory of transplantation: further evidence. *Current Opinion in Immunology*.

[13] Cai J, Terasaki PI (2005). Human leukocyte antigen antibodies for monitoring transplant patients. *Surgery Today*.

[14] Duquesnoy RJ, Marrari M, Mulder A, Claas FHJ, Mostecki J, Balazs I (2012). Structural aspects of human leukocyte antigen class I epitopes detected by human monoclonal antibodies. *Human Immunology*.

[15] Ferrari-Lacraz S, Tiercy J-M, Villard J (2012). Detection of anti-HLA antibodies by solid-phase assay in kidney transplantation: friend or foe?. *Tissue Antigens*.

[16] Bahram S, Bresnahan M, Geraghty DE, Spies T (1994). A second lineage of mammalian major histocompatibility complex class I genes. *Proceedings of the National Academy of Sciences of the United States of America*.

[17] Groh V, Steinle A, Bauer S, Spies T (1998). Recognition of stress-induced MHC molecules by intestinal epithelial *γδ* T cells. *Science*.

[18] Zwirner NW, Dole K, Stastny P (1999). Differential surface expression of MICA by endothelial cells, fibroblasts, keratinocytes, and monocytes. *Human Immunology*.

[19] Jinushi M, Takehara T, Kanto T (2003). Critical role of MHC class I-related chain A and B expression on IFN-*α*-stimulated dendritic cells in NK cell activation: impairment in chronic hepatitis C virus infection. *Journal of Immunology*.

[20] Molinero LL, Domaica CI, Fuertes MB, Girart MV, Rossi LE, Zwirner NW (2006). Intracellular expression of MICA in activated CD4 T lymphocytes and protection from NK cell-mediated MICA-dependent cytotoxicity. *Human Immunology*.

[21] Bauer S, Groh V, Wu J (1999). Activation of NK cells and T cells by NKG2D, a receptor for stress- inducible MICA. *Science*.

[22] Zou Y, Stastny P (2009). The role of major histocompatibility complex class I chain-related gene A antibodies in organ transplantation. *Current Opinion in Organ Transplantation*.

[23] Hourmant M, Cesbron-Gautier A, Terasaki PI (2005). Frequency and clinical implications of development of donor-specific and non-donor-specific HLA antibodies after kidney transplantation. *Journal of the American Society of Nephrology*.

[24] Colombo MB, Haworth SE, Poli F (2007). Luminex technology for anti-HLA antibody screening: evaluation of performance and of impact on laboratory routine. *Cytometry Part B*.

[25] Gibney EM, Cagle LR, Freed B, Warnell SE, Chan L, Wiseman AC (2006). Detection of donor-specific antibodies using HLA-coated microspheres: another tool for kidney transplant risk stratification. *Nephrology Dialysis Transplantation*.

[26] Mihaylova A, Baltadjieva D, Boneva P (2006). Clinical relevance of anti-HLA antibodies detected by flow-cytometry bead-based assays-single-center experience. *Human Immunology*.

[27] Wiebe C, Gibson IW, Blydt-Hansen TD (2012). Evolution and clinical pathologic correlations of de novo donor-specific HLA antibody post kidney transplant. *American Journal of Transplantation*.

[28] Ginevri F, Nocera A, Comoli P (2012). Post-transplant de novo donor-specific HLA antibodies identify pediatric kidney recipients at risk for late antibody-mediated rejection. *American Journal of Transplantation*.

[29] Wang D, Wu G, Chen J (2012). Predicting renal graft failure by sCD30 levels and de novo HLA antibodies at 1year post-transplantation. *Transplant Immunology*.

[30] Gîngu C, Moise A, Constantinescu I (2012). Cytotoxic antibodies monitoring in kidney transplantation—their clinical relevance and challenges. *Romanian Journal of Morphology and Embryology*.

[31] Everly MJ, Rebellato LM, Haisch CE (2013). Incidence and impact of de novo donor-specific alloantibody in primary renal allografts. *Transplantation*.

[32] Zwirner NW, Marcos CY, Mirbaha F, Zou Y, Stastny P (2000). Identification of MICA as a new polymorphic alloantigen recognized by antibodies in sera of organ transplant recipients. *Human Immunology*.

[33] Sumitran-Holgersson S, Wilczek HE, Holgersson J, Soderstrom K (2002). Identification of the nonclassical HLA molecules, mica, as targets for humoral immunity associated with irreversible rejection of kidney allografts. *Transplantation*.

[34] Li L, Chen A, Chaudhuri A (2010). Compartmental localization and clinical relevance of MICA antibodies after renal transplantation. *Transplantation*.

[35] Terasaki PI (2003). Humoral theory of transplantation. *American Journal of Transplantation*.

[36] Piazza A, Pocci E, Borrelli L (2001). Impact of donor-specific antibodies on chronic rejection occurrence and graft loss in renal transplantation: posttransplant analysis using flow cytometric techniques1. *Transplantation*.

[37] Terasaki PI, Cai J (2005). Humoral theory of transplantation: further evidence. *Current Opinion in Immunology*.

[38] Deng C-T, El-Awar N, Ozawa M, Cai J, Lachmann N, Terasaki PI (2008). Human leukocyte antigen class II DQ alpha and beta epitopes identified from sera of kidney allograft recipients. *Transplantation*.

[39] Zhang Q, Liang LW, Gjertson DW (2005). Development of posttransplant antidonor HLAantibodies is associated with acute humoral rejection and early graft dysfunction. *Transplantation*.

[40] Mao Q, Terasaki PI, Cai J (2007). Extremely high association between appearance of HLA antibodies and failure of kidney grafts in a five-year longitudinal study. *American Journal of Transplantation*.

[41] Alberu J, Vargas-Rojas MI, Morales-Buenrostro LE (2012). Donor-specific HLA antibody development and peripheral CD4^+^CD25^high^ cells in kidney transplant recipients: a place for interaction?. *Journal of Transplantation*.

[42] Lee P-C, Zhu L, Terasaki PI, Everly MJ (2009). HLA-specific antibodies developed in the first year posttransplant are predictive of chronic rejection and renal graft loss. *Transplantation*.

[43] Huang Y, Ramon D, Luan FL (2012). Incidences of preformed and de novo donor-specific HLA antibodies and their clinicohistological correlates in the early course of kidney transplantation. *Clinical Transplantation*.

[44] DeVos JM, Patel SJ, Burns KM (2011). De novo donor specific antibodies and patient outcomes in renal transplantation. *Clinical Transplantation*.

[45] Cooper JE, Gralla J, Chan L (2011). Clinical significance of post kidney transplant de novo DSA in otherwise stable grafts. *Clinical Transplantation*.

[46] Terasaki P, Lachmann N, Cai J (2006). Summary of the effect of de novo HLA antibodies on chronic kidney graft failure. *Clinical Transplants*.

[47] Zou Y, Heinemann FM, Grosse-Wilde H (2006). Detection of Anti-MICA antibodies in patients awaiting kidney transplantation, during the post-transplant course, and in eluates from rejected kidney allografts by luminex flow cytometry. *Human Immunology*.

[48] Mizutani K, Terasaki P, Rosen A (2005). Serial ten-year follow-up of HLA and MICA antibody production prior to kidney graft failure. *American Journal of Transplantation*.

[49] Zou Y, Stastny P, Süsal C, Döhler B, Opelz G (2007). Antibodies against MICA antigens and kidney-transplant rejection. *The New England Journal of Medicine*.

[50] Racusen LC, Colvin RB, Solez K (2003). Antibody-mediated rejection criteria—an addition to the Banff ’97 classification of renal allograft rejection. *American Journal of Transplantation*.

[51] Mizutani K, Shibata L, Ozawa M (2006). Detection of HLA and MICA antibodies before kidney graft failure. *Clinical Transplants*.

[52] Racusen LC (2003). Pathology and the allo-immune response. *American Journal of Transplantation*.

[53] Hidalgo LG, Campbell PM, Sis B (2009). De novo donor-specific antibody at the time of kidney transplant biopsy associates with microvascular pathology and late graft failure. *American Journal of Transplantation*.

[54] Loupy A, Suberbielle-Boissel C, Hill GS (2009). Outcome of subclinical antibody-mediated rejection in kidney transplant recipients with preformed donor-specific antibodies. *American Journal of Transplantation*.

[55] Sis B, Jhangri GS, Bunnag S, Allanach K, Kaplan B, Halloran PF (2009). Endothelial gene expression in kidney transplants with alloantibody indicates Antibody-mediated damage despite lack of C4d staining. *American Journal of Transplantation*.

[56] Sis B, Mengel M, Haas M (2010). Banff ’09 meeting report: antibody mediated graft deterioration and implementation of Banff working groups. *American Journal of Transplantation*.

[57] Böhmig GA, Exner M, Habicht A (2002). Capillary C4d deposition in kidney allografts: a specific marker of alloantibody-dependent graft injury. *Journal of the American Society of Nephrology*.

[58] Haririan A, Kiangkitiwan B, Kukuruga D (2009). The impact of C4d pattern and donor-specific antibody on graft survival in recipients requiring indication renal allograft biopsy. *American Journal of Transplantation*.

[59] Kedainis RL, Koch MJ, Brennan DC, Liapis H (2009). Focal C4d+ in renal allografts is associated with the presence of donor-specific antibodies and decreased allograft survival. *American Journal of Transplantation*.

[60] Loupy A, Cazes A, Guillemain R (2011). Performance of routine C4d and C3d immunostaining on protocol EMBs in a prospective and unselected cohort of heart transplant patients. *The Journal of Heart and Lung Transplantation*.

[61] Patel R, Terasaki PI (1969). Significance of the positive crossmatch test in kidney transplantation. *The New England Journal of Medicine*.

[62] Lefaucheur C, Loupy A, Hill GS (2010). Preexisting donor-specific HLA antibodies predict outcome in kidney transplantation. *Journal of the American Society of Nephrology*.

[63] Ting A, Morris PJ (1983). Successful transplantation with a positive T and B cell crossmatch due to autoreactive antibodies. *Tissue Antigens*.

[64] Süsal C, Döhler B, Opelz G (2009). Presensitized kidney graft recipients with HLA class I and II antibodies are at increased risk for graft failure: a Collaborative Transplant Study report. *Human Immunology*.

[65] Roelen DL, Doxiadis II, Claas FH (2012). Detection and clinical relevance of donor specific HLA antibodies: a matter of debate. *Transplant International*.

[66] Wahrmann M, Bartel G, Exner M (2009). Clinical relevance of preformed C4d-fixing and non-C4d-fixing HLA single antigen reactivity in renal allograft recipients. *Transplant International*.

[67] Amico P, Hönger G, Mayr M, Steiger J, Hopfer H, Schaub S (2009). Clinical relevance of pretransplant donor-specific HLA antibodies detected by single-antigen flow-beads. *Transplantation*.

[68] Couzi L, Araujo C, Guidicelli G (2011). Interpretation of positive flow cytometric crossmatch in the era of the single-antigen bead assay. *Transplantation*.

[69] Blume OR, Yost SE, Kaplan B (2012). Antibody-mediated rejection: pathogenesis, prevention, treatment, and outcomes. *Journal of Transplantation*.

[70] Morris GP, Phelan DL, Jendrisak MD, Mohanakumar T (2010). Virtual crossmatch by identification of donor-specific anti-human leukocyte antigen antibodies by solid-phase immunoassay: a 30-month analysis in living donor kidney transplantation. *Human Immunology*.

[71] Loupy A, Hill GS, Jordan SC (2012). The impact of donor-specific anti-HLA antibodies on late kidney allograft failure. *Nature Reviews Nephrology*.

[72] Sberro-Soussan R, Zuber J, Suberbielle-Boissel C (2010). Bortezomib as the sole post-renal transplantation desensitization agent does not decrease donor-specific anti-HLA antibodies. *American Journal of Transplantation*.

